# Qualitative assessment of providers’ experiences with a segmentation counseling tool for family planning in Niger

**DOI:** 10.1186/s12978-023-01617-9

**Published:** 2023-05-10

**Authors:** Ellen MacLachlan, Balki Ibrahim Agali, Amelia Maytan-Joneydi, Sanoussi Chaibou, Souleymane Amadou Garba, Illiassou Chaibou Halidou, Ilene S. Speizer, Abdoul Moumouni Nouhou

**Affiliations:** 1 Independent Global Health Consultant, Seattle, WA USA; 2 L’Initiative OASIS Niger, Niamey, Niger; 3 Carolina Population Center, University of North Carolina at Chapel Hill, Chapel Hill, NC USA; 4 Department of Maternal and Child Health, University of North Carolina at Chapel Hill, Chapel Hill, NC USA

**Keywords:** Family planning, Niger, Sub-Saharan Africa, Reproductive health services, Quality of counseling, Segmentation, Program scale-up, Planification familiale, Niger, Afrique sub-saharienne, Services de santé reproductive, Qualité des conseils, Segmentation, Mise à l’échelle du programme

## Abstract

**Introduction:**

This study examines experiences with a family planning segmentation counseling tool that is used during the provision of family planning services. Based on answers to a series of questions, women are segmented into one of five categories of family planning users and counseled based on their identified segment. This study aimed to qualitatively assess provider perspectives on implementation of the tool.

**Methods:**

Semi-structured in-depth interviews took place in the Dosso region of Niger among 16 family planning providers who had been trained in segmentation and were currently using the segmentation tool. The facilities chosen for interviews were part of a larger mixed methods study assessing the impact of using the segmentation approach. Interview questions focused on training, supervision, how segmentation occurs at the health facility, how segmentation changes provider–client interactions, and any difficulties faced with implementation. Interviews were translated and transcribed into French and data were coded and thematically analyzed.

**Results:**

All providers in the study reported positive outcomes associated with segmentation. While providers acknowledged that the segmentation approach added time to the clinic visit, they did see the benefit of this extra time in providing more meaningful interactions between clients and providers, leaving clients with a deeper understanding of family planning and of the different methods available. The implementation of the tool did not change other aspects of service delivery, except that a segmentation sheet was required to be filled in and kept in each patient’s file. Difficulties reported included translating the segmentation tool questions into local languages, training enough health care providers and avoiding stock outs of the segmentation sheets.

**Conclusion:**

The segmentation process is of benefit to family planning clients in Niger and the scale-up of the strategy could bring higher quality services to women. If this approach is brought to scale the implementation challenges uncovered need to be addressed, especially adequate training. Further research is needed to determine if segmentation leads to changes in family planning use outcomes.

**Supplementary Information:**

The online version contains supplementary material available at 10.1186/s12978-023-01617-9.

## Plain language summary

This is a study about what nurses and other health care staff in Niger think about a segmentation counseling tool that is used during family planning services. Segmentation means that during counseling each woman is asked a series of questions and, depending on her answers, she is segmented into one of five ‘client type’ categories and counseled based on that ‘client type’ or segment. Face-to-face interviews with health care staff took place in the Dosso region of Niger among 16 staff who were using the tool with their patients. The facilities chosen for interviews were part of a larger study assessing how well segmentation was working. Staff were asked about training, supervision, how segmentation occurs at the facility, how segmentation changes how they interact with patients, and any difficulties faced with implementation. All the interviews were then analyzed. All providers in the study had positive things to say about segmentation, including the benefit of spending extra time with each client. This extra time results in more meaningful interactions between clients and providers, leaving clients with a deeper understanding of family planning and of the different methods available. Difficulties reported included translating the segmentation tool questions into local languages, training enough health care providers and avoiding stock outs of the segmentation sheets. The segmentation process is of benefit to family planning clients in Niger and can contribute to higher quality family planning services for women. The challenges that were found need to be addressed, especially adequate training.

**Supplementary Information:**

The online version contains supplementary material available at 10.1186/s12978-023-01617-9.

## Résumé

**Introduction:**

Cette étude examine les expériences d’un outil de conseil de segmentation en planification familiale utilisé lors des prestations de services de planification familiale. Sur la base des réponses données à une série de questions, les femmes sont réparties dans l’une des cinq catégories d’utilisatrices de planification familiale (segment) et conseillées en fonction de leur segment identifié. Cette étude vise à évaluer qualitativement les perceptions des prestataires sur l’utilisation de l’outil de conseil de segmentation.

**Méthodes:**

Des entretiens approfondis semi-structurés ont été effectués dans la région de Dosso au Niger auprès de 16 prestataires de planification familiale formés à l’utilisation de l’outil et l’utilisant au moment de la collecte des données. Les structures de santé sélectionnés pour les entretiens figurent dans une étude plus vaste à méthodes mixtes évaluant l’impact de l’utilisation de l’approche de segmentation. Les questions d’entretien portaient sur la formation, la supervision, la façon dont la segmentation se produit dans l’établissement de santé, comment la segmentation modifie les interactions prestataire-client et toutes les difficultés rencontrées lors de la mise en œuvre. Les entretiens ont été traduits et transcrits en français et les données ont été codées et analysées par thématique.

**Résultats:**

Tous les prestataires de l’étude ont rapporté des résultats positifs associés à la segmentation. Bien que les prestataires aient reconnu que l’approche de segmentation a ajouté du temps à la visite au centre de santé, ils ont vu l’avantage de ce temps supplémentaire en fournissant des interactions plus significatives entre les clients et les prestataires, laissant aux clients une meilleure compréhension de la planification familiale et des différentes méthodes disponibles. La mise en place de l’outil n’a pas changé les autres aspects de la prestation de services, si ce n’est qu’une fiche de segmentation devait être remplie et conservée dans le dossier de chaque patient. Les difficultés signalées comprenaient la traduction des questions de l’outil de segmentation dans les langues locales, la formation d’un nombre suffisant de prestataires de soins de santé et la prévention des ruptures de stock de fiches de segmentation.

**Conclusion:**

Le processus de segmentation profite aux clientes de la planification familiale au Niger et la mise à l’échelle de la stratégie pourrait apporter des services de meilleure qualité aux femmes. Si cette approche est portée à grande échelle, les défis de mise en œuvre découverts doivent être relevés, en particulier une formation sur l’utilisation de l’outil adéquate. Des recherches supplémentaires sont nécessaires pour déterminer si la segmentation entraîne des changements dans les résultats de l’utilisation de la planification familiale.

## Introduction

Niger has long been recognized as a country with high fertility and low contraceptive use. The 2012 Demographic and Health Survey estimated that the total fertility rate was 7.6 births per woman [1]. In 2017, the Performance Monitoring for Action (PMA) project estimated that the modern contraceptive prevalence in Niger was 15.2% among all women and 18.1% among women married or in union [2]. PMA also found that only 27% of women in union were visited by a health worker or visited a health facility and discussed family planning with a provider [2]; this suggests gaps in family planning awareness for women who may need information to help them to delay or avoid childbearing. The Government of Niger has made significant commitments as part of their engagement in the FP2020 initiative to increasing contraceptive use to help improve the health and well-being of women throughout the nation. In 2012, the government set an ambitious goal of attaining a modern contraceptive prevalence of 50% by 2020; the estimated 2020 modern contraceptive prevalence level was 16.9% [3]. Even though the country did not meet the 2020 goal, in October of 2021 Niger set a new goal of attaining a modern contraceptive prevalence of 36.8% by 2030 as part of FP2030 [4].

Ensuring high quality health services is not only a human right but also important for ensuring that all women, men, and children are able to attain their highest potential in terms of health and well-being [5]. Numerous strategies have been implemented to improve the quality of family planning (FP) services provided, including whole-site training, clinic makeovers, commodity security strategies, task shifting, integration of family planning with other maternal and child health services, and both clinical and counseling training of health care providers [6–9]. One strategy initially launched in the late 1990s by the Population Council is the Balanced Counseling Strategy (BCS). BCS is a strategy whereby providers are trained on how to counsel clients based on the clients’ specific needs to support informed FP decision-making [10]. The BCS approach is meant to address some of the key elements of quality of care identified by Judith Bruce in 1990, including choice of methods, information provided to clients, interpersonal relations, and follow-up and continuity mechanisms [11]. BCS was found to lead to improved quality of services and was associated with increases in clients’ knowledge and use of effective methods of family planning [12, 13].

In Niger the government has identified a segmentation counseling strategy that it intends to scale-up; this segmentation approach is similar to BCS because it identifies categories of women and trains providers to provide counseling specific to each group’s needs [14, 15]. The Government of Niger is employing the segmentation strategy to help increase contraceptive use. Through segmenting clients, it is hypothesized that all women visiting a family planning provider will be offered high quality services, including being introduced to a range of methods to meet their current family planning needs. Market, or audience, segmentation for family planning is an approach that has been used for decades to improve the quality of FP services [16].

The segmentation strategy in Niger was developed through a rigorous process that included qualitative and quantitative data collection from 2013 to 2014. Focus group discussions with married and unmarried women and men aged 15–24 and 25 and over, in-depth interviews with FP providers, and observations of FP consultations were conducted. The results of the qualitative data analysis were used to develop initial segments. Next, over 2000 women, from a nationally representative sample in Niger, were surveyed [17]. The quantitative data were filtered using key variables, resulting in the identification of five distinct segments of women for the Niger context—Healthy Proactives, Traditional Autonomists, Sheltered Skeptics, Modern Elites, and Conservative Passives (Additional file 3: Figure S1). A similar approach has been used to identify health care provider segments in Burkina Faso, Pakistan and Tanzania as part of the Beyond Bias project [18]. The family planning segments are based on key behaviors and attitudes that relate to contraceptive use and counseling needs by group such as openness to modern methods, influence of religion, and desired family size. For each of the segments, specific counseling tools were developed to inform counseling around family size desires and appropriate methods to meet stated needs. A segmentation tool was designed with twelve questions related to a client’s family planning experiences and expectations (Additional file 3: Figure S1) that are asked by the provider to all women seeking family planning services at a health facility. Based on the answers to these questions, women are categorized into one of the five categories and counseled based on their identified category [15]. While asking the 12 questions adds time to a client visit, the counseling is somewhat more targeted and may eventually lead to less time spent during the visit. This strategy was first introduced into the Niger healthcare system in collaboration with Pathfinder International in 2017.

In the initial testing of the segmentation approach in Niger, Pathfinder International found that providers found it easy to use, were able to use it, and it was associated with method adoption [17]. Based on these findings, segmentation is considered a promising strategy that is meant to optimize the time that providers spend with clients and improve the services that they receive during a family planning visit. Recommendations from the pilot were that segmentation should be scaled up, however, before taking it to scale, the Government of Niger and Pathfinder International deemed it necessary to undertake a mixed methods evaluation of the strategy. This paper reports the qualitative findings from health care providers about their experiences with segmentation at facilities where segmentation has been implemented; this information is important for informing potential scale-up of the approach. In an earlier paper using exit interview data with clients from the same study sites in Niger, it was found that clients reported higher quality of care in facilities that had segmentation than in facilities without segmentation and those clients who were segmented reported better quality than those clients who were not segmented [19].

## Methods

### Setting

This study was part of a larger assessment of the segmentation counseling strategy that included data collection in 45 Centres de Santé Integré (CSI), or type 1 and 2 level health centers, in the Boboye, Dosso, Doutchi, Falmey, Loga, and Tibiri health districts of Dosso region of Niger. Dosso is one of eight regions in Niger and is in the extreme southwest of the country bordering Benin and Nigeria (Additional file 4: Figure S2). The Dosso region had a total population of 2,459,812 people in 2017 and is the historic center of the Dosso Kingdom [20]. Dosso region is predominately rural, with only 8.9% of the population living in urban settings in 2017 [20].

### Study design

The parent study for the data reported here comprised of a mixed methods quasi-experimental post-test only evaluation study implemented in the Dosso region between February and March 2020. The design of the parent study included three arms, two that included segmentation and one that did not include segmentation (the comparison arm). For the study presented here, the qualitative portion of the mixed methods evaluation, 16 of the 30 CSIs in the implementation arms of the parent study were selected to participate in qualitative data collection with providers. In each of the 16 CSIs, one health care provider was invited to participate in an in-depth interview (IDI) if they had been trained on segmentation. See Additional file 5: Figure S3 for visual presentation of the study design for the qualitative portion of the study.

### Interview guide development

A qualitative IDI interview guide was developed to explore healthcare provider experiences and perspectives on the segmentation tool. The IDI interview guide was divided into two parts. Part 1 included two vignettes about hypothetical family planning clients asked in all arms. Data from Part 1 is not included in this paper. Part 2 of the guide included questions about use of the segmentation tool at the CSI. Data drawn from questions in Part 2 of the guide are presented in this paper. Part 2 of the interview guide was divided into these topic areas: (1) training on the segmentation tool, (2) experiences with using the segmentation tool, (3) influence of the segmentation process on family planning methods offered by the provider or chosen by the client, and (4) general feedback on segmentation. Each of the questions in the guide included probes to draw out more rich information about providers’ use of the segmentation tool. The interview guide was piloted with four health care providers outside the study area before data collection began to ensure the questions were appropriate; all questions were refined based on feedback during the pilot. During training of interviewers, the team discussed the translation of each question into Zarma and Hausa since these are generally not written languages. The final interview guide is provided in the Supplement.

### Data collection

In-depth interviews were conducted in private rooms at the CSIs where the chosen provider was located. The entire in-depth interview ranged in length from 32 to 100 min. One female or one male trained interviewer conducted the IDIs in Zarma, Hausa, and French. All interviewees provided demographic information regarding their gender, age, health care cadre, training and education, number of years working at the facility and number of years offering family planning services. All interviews were audio recorded after obtaining written consent from the providers. The Zarma and Hausa audio recordings were first translated and then transcribed into French and any French audio recordings were directly transcribed. The final de-identified French transcriptions were uploaded onto a web-based collaborative qualitative research analysis platform called Dedoose [21]. Analysis was undertaken in French and the quotes presented in this paper were translated into English by our Niger study team and checked for clarity by our USA study team.

### Data analysis

Preliminary codebooks for Part 1 and Part 2 of the guide were developed based on both the questions in the interview guide and a review of the content in three randomly selected interview transcripts. The codebooks contained both major themes called ‘parent’ codes and sub-themes called ‘child’ codes. These codes were then entered into Dedoose. Four investigators then coded the first six interviews using the parent codes, compared their results, and revised the parent codes in the codebooks. The same four investigators (SC, AM, BA, IC) then coded the ten subsequent interview transcripts using these parent codes while continuing to reach consensus on the definitions and distinct meanings of each parent code. Once parent coding was complete, two investigators (AM, BA) coded the segmentation child codes for all 16 interviews using the child codes in the codebook, revising the codebook as needed based on reaching consensus of code definitions. Inter-rater reliability (IRR) testing was conducted once for the four coders who coded parent codes and twice for the two coders who coded child codes, both compared to a master coder (EM), as an additional measure of coding quality. The IRR tests were conducted in Dedoose. Dedoose code-specific application results are reported using Cohen’s kappa statistic [22, 23]. Analysis consisted of a thematic analysis that examined content under each parent code and its associated child codes, focusing on the objectives of the study and differences by descriptors for each theme. All coders reached consensus during the final stages of analysis on the themes that emerged from the coded data.

## Results

A total of 16 health care providers participated in the study, with a 100% participation rate. The 16 providers interviewed had all been trained in segmentation and had been using the segmentation tool for a mean of 16 months (range 1–60 months). Although the plan was to include eight providers from each arm, in the end, interviews were collected from seven providers from Arm 1 of the study and nine providers from Arm 2. The providers represented four distinct roles at the CSIs: Chief of the CSI (3 providers), Deputy Chief of the CSI (3 providers), Nurse (5 providers), and Midwife (5 providers). The mean age of the providers was 38 years and they were predominately female (81%). Half of the 16 interviews were with providers from Boboye health district, with the rest of the interviews represented by Dosso (3 interviews), Doutchi (3 interviews) and Loga (2 interviews) health districts. The mean number of years providing family planning services was eight years, with a range from 1 to 28 years. There were two types of trainings offered in segmentation, either a full five-day training or an on-site briefing at the CSI by either another CSI staff person who had attended the full training or by someone associated with Pathfinder International. In total seven of those interviewed had received the full five-day training in segmentation and six had received a briefing. Three providers did not specify the type of training received. See Additional file 1: Table S1.

All four coders involved in coding the segmentation parent codes had an average Cohen’s kappa score of 0.79 and a range of 0.72–0.89, when each was compared to a master coder. The two child code coders had an average Cohen’s kappa score of 0.69 for coding of the segmentation codes when compared to a master coder (see Additional file 2: Table S2). All these scores show substantial agreement (between 0.61 and 0.80 kappa score) and two show almost perfect agreement (between 0.81 and 1.00 kappa score) [23].

### Training

Since all 16 interviewees had received either the five-day or brief training on the segmentation tool, they were asked about what the content of the training had been to gauge their comprehension of content and experiences across the two training formats. Responses from providers about their training was important because training was a prerequisite for providers to use the segmentation tool and provider responses reflect and verify that they had received the training content needed to make them comfortable using the segmentation tool.

A total of 15 respondents provided information on the content of their training. All respondents reported that the segmentation training, in whatever format, mainly focused on how to implement the segmentation tool and the accompanying counseling cards.

*“She just explained to us a template that she used. How we ask a woman questions, and how we take notes from the answers… at the end you check the number, you circle the greatest number, then you check the total. Based on this, you report what is written on the sheet. For example, if it is in TA [Traditional Autonomist], you report TA on the form. Checking the card, you will see* Traditional Autonomist*. This is what she showed us”.*

**-Deputy Chief who received a briefing on segmentation**

A little over half of the respondents reported that the training included content on best practices for counseling using the segmentation approach, though many respondents did not mention counseling as a specific content area.

Other content reported was a brief overview of family planning technologies and reproductive health and time spent translating the tool from French into local languages. Generally, the full trainings were the most well received.

*“We were trained in Dosso, we were trained on the segmentation strategy, on how to assess women’s knowledge about FP [Family Planning], how to classify a woman regarding her knowledge. During the training, we had no problems, the trainers were really impeccable because they trained us very well, we learned a lot from them.”*

**-Chief who attended full segmentation training**

A total of ten respondents provided information on the type of additional content they would desire in a training. A main theme that emerged among respondents was a desire to have more in depth training on segmentation. This was reported especially from those health care providers who had been briefed on segmentation by the Pathfinder International team instead of attending a full five-day training.

*“What I want is that my capacities regarding the segmentation strategy be strengthened. I need training on that.”*

**-Midwife who received a briefing on segmentation**

### Supervision

Eleven of the 16 respondents confirmed that they had received a supervision visit after the training. Questions about supervision were included because supervision visits were meant to support providers in the use of the segmentation counseling tools and helped refresh previously-trained providers on the use of the segmentation tools and gave an opportunity for a briefing of untrained providers on how to use the tools. Supervision visits happened through both planned and unplanned visits to the facility. Respondents reported that the supervision visits included members of the Pathfinder International team, health district agents and agents from the regional public health directorate (Direction Régionale de la Santé Publique). In addition to a review of segmentation procedures, most of the providers reported that the supervision team reviewed the CSI’s data in terms of the proportion of clients segmented.

*“Yes, there was a visit during which, when the team came, they took the cards to count the number of women segmented and those who are not. Then they did the percentage calculation. This is what they did in addition to checking the product inventory. Then they asked us questions about what we need to be clarified.”*

**- Deputy Chief who received a briefing on segmentation**

Eight providers, of the 11 who had stated that they had received a supervision visit, provided reflections on the supervision visits. Five of these eight providers reflected that the supervision visit helped them with segmentation.

*“This benefited me because there are parts where I did not understand anything about the questionnaire during the training, because the trainers are Hausa. But when the supervision came, they explained it to me and I understood.”*

**-Midwife who received a briefing on segmentation**

### Process of implementation

During the interviews, the 16 providers were asked about the process used in their CSI to segment clients. Providers reported that first, when a client arrives at the clinic, they welcome and register all clients as they normally would, asking the reason for their visit. When the client begins a family planning consultation, the providers check a woman’s family planning file to see if the segmentation tool has been filled out and filed previously and, if not, they proceed to segmentation. Half of the providers responded that they segment only new clients to the CSI but many said they also segment returning clients if they have not yet been segmented. The rest did not specify.

*“Anyone that comes has a card. For example, this one is an old one, she has a card and has never been segmented. We segment her and we mention it on the card. If it's a first visit case, we also segment her and mention it on her card.”*

**-Midwife who received a briefing on segmentation**

All providers said that they use the segmentation tool with every kind of family planning client—young, married, unmarried, multiparous and nulliparous.

“*Interviewer: For all clients that come, do you need to do the segmentation for them or not?”*

*“Provider: Me, for all women that come, I do the segmentation.”*

*“Interviewer: And the counseling cards, do you use them with all women or not?”*

*“Provider: I use it with all women.”*

**-Chief who attended full segmentation training**

The steps of the segmentation process during a clinic visit were explained fully by a few providers, especially for new family planning clients.

*“After that I’m going to record the woman, give her a number like she is really new. I’m going to give her a FP card, this woman should be segmented, we must segment her, ask her questions. Now after having segmented her, we will see in which group she falls and we will check and see the kind of advice that the girl needs to give her according to the different segments, if she is a traditional autonomous, a conservative passive or something like that ...”*

**-Deputy Chief who received a briefing on segmentation**

### Impact of segmentation

The 16 providers interviewed were asked about the way in which segmentation changed the family planning services they offer. All the providers except for one responded that the use of the segmentation tool increased the amount of time needed for the delivery of family planning services. Respondents said this extra time allowed them to provide more information about family planning to their clients and led to more individualized counseling.

“*Interviewer: Are the questions you are asking, are they taking longer now than when there was no segmentation?*”

“*Provider: We need much more time now. Because, it was just the method before. But now, in addition to the method, there is segmentation, so it takes more time.*”

**-Midwife who received a briefing on segmentation**

*“It allows you to interact more with the client and spend more time. The client becomes comfortable and trust develops between you.”*

**-Nurse who did not specify the type of training received**

Nevertheless, the extra time needed to implement the tool was sometimes a challenge for providers. For example, a few providers said they could not implement the tool on market days due to the number of women seeking family planning on those days and the amount of time it would take to segment them all. However, some providers said that the segmentation tool became easier to use with time and did not take as much time as at the beginning.

*“There is only one difficulty when you are at the beginning of its use, even being too slow with the questions asked to women at the beginning, they think that they are wasting their time, is already a difficulty. But over time, if the health provider gets used to it, there will come a time when he doesn’t have to look at the grid to be able to ask questions, and he can finish it in two minutes.”*

**-Deputy Chief who received a briefing on segmentation**

The providers explained that the clients who had been segmented had a better understanding of family planning, of the utility and importance of family planning, and had more knowledge of different family planning methods and how they worked.

*“There is really a difference, because now you take your time, before doing FP, you make it very clear to the woman by explaining to her in her language, she answers you and you take note and after that you take the advice card, you explain to the woman how things work… now with segmentation you are obliged to go step by step so that the woman understands, and with the advice card you explain again to the woman. There are women who say, at the end of their counseling, that they are able to explain to other women who don’t understand FP.”*

**-Deputy Chief who received a briefing on segmentation**

*“And usually, women receive much more information, even regarding the method they are using. They are using these methods, but before, it’s the system that doesn’t allow us to give all the information about the methods.”*

**-Chief who attended full segmentation training**

Half of the providers stated that women learned about the segmentation approach from other women in their communities and their interest in family planning grew from there. A few providers noted that segmentation reduced beliefs in rumors about family planning. One provider gave an example of a client who asked why she was not segmented during her visit (the provider said she was too busy to segment that day) so the client came back another day to be segmented and learn about the methods available to her.

“*Interviewer: Has it [segmentation strategy] improved or disadvantaged the FP service you offer?*”

“*Provider: It has improved the service.*”

“*Interviewer: how?*”

“*Provider: Before, clients did not come abundantly for FP; but now, due to the conversations, we explain to them and they understand the usefulness of family planning. There are many to come.*”

**-Nurse who did not specify the type of training received**

*“Provider: First, with the old counseling method, where there was no segmentation, it was not possible to explain much to the woman the importance of FP, whereas with segmentation, with the questions that we ask the woman, we understand to which segment she belongs, and this helps to better sensitize her so that she accepts FP better, but also that she makes others accept as well. We transform her into a kind of relay so that she sensitizes other women to use FP. So, when a woman comes here, a hesitant woman, or a woman who does not have too much confidence, after the segmentation, we are sure that she is 100% confident in using FP, but also, she can ensure that other women also trust FP.”*

**-Chief who attended full segmentation training**

### Difficulties with segmentation

Fourteen of the 16 respondents who had experience with using the segmentation tool provided insight into the most common difficulties they faced when using the tool. The respondents indicated that there were three main themes that emerged regarding difficulties with the tools: lack of comprehension of the questions by the clients, difficulties in translating the questions into the local languages and the need for further training in segmentation (see Training above).

Providers noted that clients often had a lack of comprehension of the segmentation questions, which meant that the provider had to re-explain the question several times in the local language before the client fully understood. In many cases the provider was translating directly from French into the local language.

*“Interviewer: What are the difficulties you encounter in using the segmentation tool?”*

*“Provider: Sometimes it’s the questions, because at least and fortunately for others there is the translation into Zarma, but when it’s in French how to translate on the spot by asking the questions, sometimes it’s really difficult. In any case, that’s the difficulty, translating the questionnaire into Zarma, the client may not understand what you really mean, you have to explain the question again, that's my great difficulty.”*

**-Midwife who received a briefing on segmentation**

The lack of comprehension of questions in the tool was closely linked with difficulties associated with translating tool questions from French into the local language. This was another main difficulty that emerged from the interviews. However, it did seem that this was a difficulty that improved over time.

*“Well, when I did the training on the segmentation tool, everything is in French, we translated that afterwards into Hausa and I mastered it little by little, with the learning and the proof is that you see that sometimes I try to explain some to you without the card.”*

**-Nurse who attended full segmentation training**

## Discussion

In this qualitative sub study, part of a larger assessment of FP segmentation counseling in Niger, the majority of health care providers interviewed found the client segmentation process to be of benefit to women seeking family planning. When health care providers use the segmentation tool in their health centers, while it initially takes more time to implement the 12 questions in the tool, over time the providers become more efficient at asking the questions and the time needed to implement the tool decreases. Further, by segmenting the clients, the providers have more time to discuss targeted family planning messages with their clients and can adjust the discussion more closely to the client’s family planning needs. The in-depth counseling that accompanies segmentation allows women to better understand the different methods available and increases their confidence that they, and other women in their community, should use family planning. Such in-depth counseling is vital to family planning service provision [24–27], and is consistent with findings from other studies that use the Balanced Counseling Strategy (BCS), akin to segmentation, to improve family planning service provision. These studies similarly find significant enhancement of quality of care, an increase in session length, and improved client knowledge of the method chosen when BCS is used [28–32]. Although many reports of market segmentation in FP exist [33–35], we could not find reports of the use of these market segments by health care providers for FP counseling, such as we report here.

Although the implementation of the tool had some identified bottlenecks, such as stock outs of the segmentation sheet, inadequate training of providers, and translation issues, all providers agreed that the segmentation and counseling cards greatly improves the quality of family planning services they offer. These findings are timely for the Government of Niger as they consider supporting scale-up of the segmentation strategy nationwide. Central to this scale-up process, and all scale-up processes, is ensuring quality of services and this is where the segmentation tool can be of great use [36–38]. If the implementation issues can be systematically addressed during scale-up of this approach, the impact on family planning services in Niger could be manifold.

It is clear from the responses that the segmentation trainings, whether the 5-day training or the briefings, gave health providers the basic information needed to implement segmentation-based family planning services and should be scaled up. The quote shown from the provider who was briefed in segmentation shows a detailed understanding of how the segmentation tool is meant to be used, including exactly how the final segment is determined. Other training topics, such as best practices for counseling, family planning technologies and tool translation, were much less commonly reported, especially by providers who were briefed. This indicates the main drawback of only briefing providers on segmentation; there is a lost opportunity for a more in-depth and nuanced understanding about the rationale for segmentation and for deeper engagement on other relevant family planning updates.

One way to support CSIs in their segmentation activities is to assure adequate supervision after training. In our study, eleven of the 16 providers had received a supervision visit after training and this proportion could be increased to 100%. The responses from those interviewed clearly indicate that the supervision visit was in fact an important opportunity for further coaching and support on segmentation implementation and not just a visit for stock checking and data collection. The example given of a provider who was given assistance in interpreting tool questions during a supervision visit was a typical experience.

The process of implementation, the changes in health service delivery and the impact of segmentation on family planning clients were the most noteworthy findings from our study. The process of implementation analyses showed very few changes to the typical process of providing services to family planning clients, which means that scaling up this strategy will mean just slight changes to CSI service delivery (e.g., keeping the segmentation sheet in a client’s health care file folder). We found that all providers segment new clients and some segment returning clients if they have not been segmented previously, and that clients of all types and ages are segmented. However, this information was only qualitatively determined. Future studies of segmentation may want to include quantifiable data on the number and types of women segmented and changes in segmentation rates longitudinally from the time of introduction and onward. This could help to understand the process of implementation more fully than what is available from qualitative data.

When asked about how the segmentation process influenced family planning service delivery, the providers offered rich information about how they felt it changed their work and how they believed it impacted the clients themselves. Almost all providers reported that segmentation increased the amount of time they spent in consultation with a family planning client, allowing them to better explain the different methods and to better understand the client’s background and needs. As noted, this aspect of counseling is an essential component of quality family planning services [24–27]. It may be that the process of segmentation, and the questions included in the tool, are less important than the extra time and attention paid to clients as a result of segmentation. This question would need to be explored with more rigorous research but clearly, regardless of the mechanism, we found evidence that the segmentation process results in more individualized family planning services during which women learn much more about family planning methods and why family planning is important than during pre-segmentation counseling sessions. We also document provider beliefs that segmentation attracts more family planning clients and that segmentation ultimately decreases family planning rumors. These two findings would need more data and research to be fully verified, but it is likely that family planning demand could grow under segmentation. Notably, due to rapid population growth, the number of women in Niger is increasing very quickly and coupled with higher FP demand, it is possible that a capacity problem may arise in the early years of scaling up segmentation.

As noted above, the process of implementation was not without difficulties. The task of translating the segmentation tool questions from French into local languages was a main challenge for providers, a process that often started during the training but continued well into the introduction of the tool at the CSIs. When providers started using the questions that had been translated into a local language, often the clients did not understand the translated question and the provider revised the translations yet again. Given the significant challenges with translation, it may be best for implementers to pilot and finalize translated tools in all local languages well before starting the training of providers, including piloting the tools with clients who speak the local language.

Another difficulty reported by providers were stock outs. Stock outs of the segmentation sheet were common and resulted in temporary halts of segmentation activities at the CSIs, since the sheets list the questions and are essential to identification of the client’s segment. Preventing stock outs of the segmentation sheets, and less commonly the counseling cards, should be prevented as much as possible during implementation to ensure continuation of the program. There may be innovative ways to assist providers in accessing the tool and avoiding stock outs, such as including the tool as part of the Ministry of Health’s vital commodities for health centers. The other important challenge was the need for comprehensive training of providers that has already been noted. Providers also encouraged that more providers are trained at facilities and not just one per facility; this will ensure that all clients are able to be segmented and not just those who come on a day when a provider trained in segmentation is present.

### Limitations

The characteristics of the interviewers may have influenced the results since they were not gender- or age-matched with interviewees. The interviewees may also have believed that the interviews were part of a performance evaluation and changed their responses to be more positive, although it was made clear during the informed consent process that this was not a consideration. This study was based on in-depth interviews with 16 health care providers. Although the 16 providers varied considerably in gender, age, position at the CSI, and other characteristics, the study could have been improved with a larger number of participants, even though when reviewing the codes we did feel saturation was reached after 6–8 interviews [39]. The number of interviews may have especially influenced themes where only a subset of the 16 participants provided responses (e.g., supervision). The inter-rater reliability (IRR) scores in the study showed substantial agreement but there still could have been coding errors occurring that may have influenced the results of this study [22]. This is particularly true for the more complex and numerous child codes used for finer content analysis of segmentation themes, where the Kappa score from IRR testing was 0.69 on average. We worked to reduce such errors as much as possible through numerous discussions of coding applications between the master coder and the two other segmentation child code coders. The results of such discussions included changes to the code definitions, recoding of applied codes and additions, reductions or merging of codes. Another limitation in the study is the fact that five of the providers, among those who reported the type of training, had received a briefing instead of a full 5-day training in segmentation. Although it is evident that these providers learned how to conduct segmentation “on the job,” the lack of formal training in segmentation may have influenced the depth and sophistication of the responses to our questions about segmentation implementation. However, this may reflect more real-world implementation of the program.

## Conclusions

Providers’ reports indicate that the segmentation process improved the quality of family planning services in their respective CSIs. The questions in the segmentation tool encourage longer consultations with women, during which women gain a more in depth understanding of the need for family planning and of the different family planning methods available. Providers reported that over time, as they became more acquainted with the tool, the time required for use of the tool became less. As found in other studies that employ segmentation-based counseling, this study shows that the segmentation strategy improves the quality of family planning services in Niger, where family planning use is low and fertility rates are high [12, 13], [28–32]. More research is needed to understand the exact mechanisms by which the segmentation process achieves this positive result in Niger and studies are needed to determine whether segmentation increases the number of family planning clients, affects continuation of a method among those who adopt, and reduces incorrect rumors about family planning methods. If segmentation is brought to scale in Niger, it is important to address implementation issues having to do with translation of the tool, stock outs of segmentation sheets and ensuring comprehensive training of one or more health care providers in each facility. Training additional providers could help ensure that all women seeking family planning benefit from segmentation and would lead to improved family planning services for all clients.

## Supplementary Information

**Additional file 1.** **Table 1.** Demographic Characteristics of Health Care Providers Participating in In-Depth Interviews regarding Segmentation in 16 Health Facilities in Niger (N = 16).

**Additional file 2.** **Table 2.** Inter-rater reliability (IRR) scores using Cohen’s kappa for parent codes and child codes (for segmentation) used in the study.

**Additional file 3.** **Figure 1.** Segmentation counseling tool developed by (removed for anonymity) for use in Niger.

**Additional file 4.** **Figure 2.** Dosso Regio of Niger.

**Additional file 5.** **Figure 3.** Data collection plan for qualitative study in the intervention arms, in Dosso region.

## Data Availability

The datasets generated and/or analyzed during the current study are not publicly available to protect the identities of the participants involved but are available from the third author on reasonable request.

## Abbreviations

BCS: Balanced Counseling Strategy
CSI: Centre de Santé Integré
FP: Family planning
IDI: In-depth interview
IMPACT: Initiative de Mobilisation pour l’Accès à la Contraception pour Tous
IRR: Inter-rater reliability
PMA: Performance monitoring for action
USA: United States of America
