# Supplementary material for: Qualitative assessment of providers’ experiences with a segmentation counseling tool for family planning in Niger
Source: Reprod Health. 2023 May 10;20:71. doi: 10.1186/s12978-023-01617-9 (PMC10170744; doi:10.1186/s12978-023-01617-9)
Supplement: Supplementary file 1 — Additional file 1. Table 1. Demographic Characteristics of Health Care Providers Participating in In-Depth Interviews regarding Segmentation in 16 Health Facilities in Niger (N = 16). [file 12978_2023_1617_MOESM1_ESM.docx]

**Table 1:** Demographic Characteristics of Health Care Providers Participating in In-Depth Interviews regarding Segmentation in 16 Health Facilities in Niger (N=16)

| **Participant Role at CSI** | **Arm** | **Gender** | **Age** | **District** | **Time Using Tool in Months** | **Years Providing FP Services** | **Type of Training in Segmentation** |
| --- | --- | --- | --- | --- | --- | --- | --- |
| #1. Nurse | 1 | F | 30 | Dosso | 36 | 3 | Not Specified |
| #2. Dep. Chief | 1 | F | 31 | Doutchi | 6 | 12 | Briefing |
| #3. Chief | 1 | M | 45 | Loga | 60 | 18 | Not Specified |
| #4. Nurse | 1 | F | 30 | Loga | 36 | 8 | Not Specified |
| #5. Midwife | 2 | F | 30 | Boboye | 2 | 5 | Briefing |
| #6. Midwife | 2 | F | 35 | Boboye | 3 | 9 | Full 5 Days |
| #7. Midwife | 2 | F | 28 | Boboye | 1 | 1 | Briefing |
| #8. Midwife | 2 | F | 33 | Boboye | 6 | 7 | Briefing |
| #9. Chief | 2 | F | 54 | Boboye | 5 | 28 | Full 5 Days |
| #10. Nurse | 2 | F | 55 | Boboye | 2 | 10 | Briefing |
| #11. Nurse | 2 | F | 35 | Boboye | 6 | 3 | Full 5 Days |
| #12. Midwife | 2 | F | 31 | Dosso | 27 | 10 | Full 5 Days |
| #13. Nurse | 2 | F | 59 | Doutchi | 30 | 6 | Full 5 Days |
| #14. Dep. Chief | 2 | F | 31 | Doutchi | 2 | 11 | Briefing |
| #15. Dep. Chief | 1 | M | 36 | Dosso | 36 | 3 | Full 5 Days |
| #16. Chief | 2 | M | 39 | Boboye | 5 | 1 | Full 5 Days |
| **19% Chiefs, 19% Deputy Chiefs, 31% Nurses, 31% Midwives** | **31% in Arm 1**  **69% in Arm 2** | **19% M**  **81% F** | **Mean**  **of 38 yrs old** | **19% Dosso, 19% Doutchi, 12% Loga, 50% Boboye** | **Mean of 16 Months (Range 1-60)** | **Mean of 8 Years (Range 1-28)** | **19% Not Specified, 38% Briefing, 43% Fully 5 Days** |

Abbreviations: CSI, Centres de Santé Integré; FP, Family Planning; Dep., Deputy; F, Female; M, Male
