# Supplementary material for: Qualitative assessment of providers’ experiences with a segmentation counseling tool for family planning in Niger
Source: Reprod Health. 2023 May 10;20:71. doi: 10.1186/s12978-023-01617-9 (PMC10170744; doi:10.1186/s12978-023-01617-9)
Supplement: Supplementary file 2 — Additional file 2. Table 2. Inter-rater reliability (IRR) scores using Cohen’s kappa for parent codes and child codes (for segmentation) used in the study. [file 12978_2023_1617_MOESM2_ESM.docx]

**TABLE 2.** Inter-rater reliability (IRR) scores using Cohen’s kappa for parent codes and child codes (for segmentation) used in the study

|  | **Parent Code Kappa Score (w/Master Coder)** | **Child Code Kappa Score**  **(w/ Master Coder)** |
| --- | --- | --- |
| **Coder #1** | 0.72 | --- |
| **Coder #2** | 0.73 | --- |
| **Coder #3** | 0.89 | 0.75 |
| **Coder #4** | 0.82 | 0.63 |
| ***Average IRR***  ***(Cohen’s kappa)*** | **0.79** | **0.69** |
