# Supplementary figures and images for: Qualitative assessment of providers’ experiences with a segmentation counseling tool for family planning in Niger
Source: Reprod Health. 2023 May 10;20:71. doi: 10.1186/s12978-023-01617-9 (PMC10170744; doi:10.1186/s12978-023-01617-9)

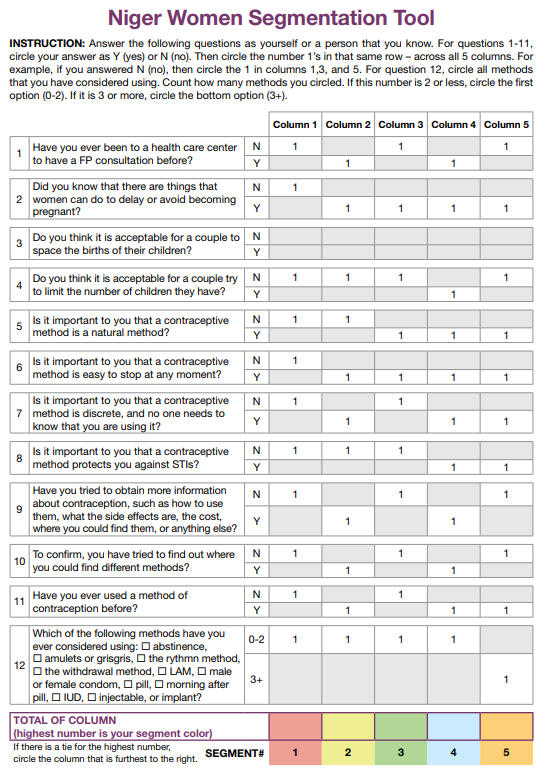

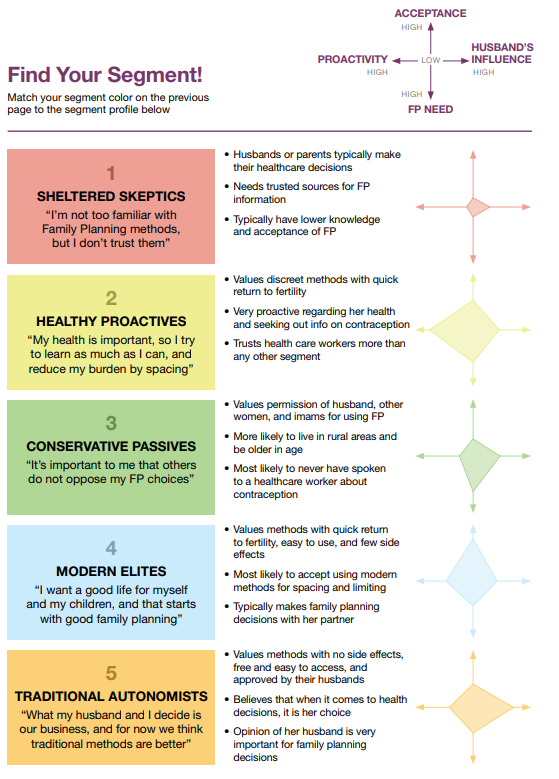
**Figure 1: Segmentation counseling tool developed by (removed for anonymity) for use in Niger**

Supplement: Supplementary file 3 — Additional file 3. Figure 1. Segmentation counseling tool developed by (removed for anonymity) for use in Niger. [file 12978_2023_1617_MOESM3_ESM.docx]

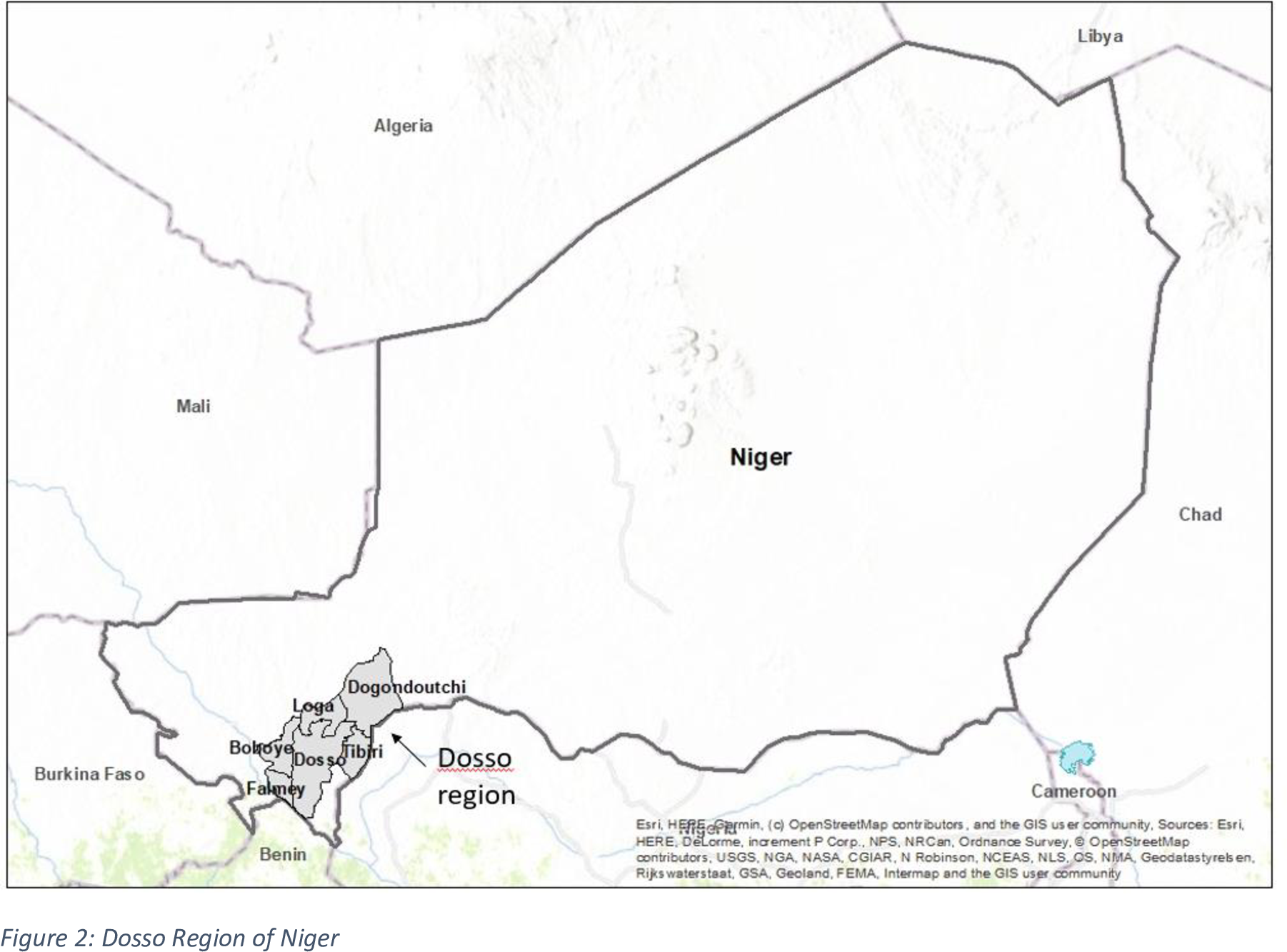

Supplement: Supplementary file 4 — Additional file 4. Figure 2. Dosso Regio of Niger. [file 12978_2023_1617_MOESM4_ESM.tif]
