# Supplementary material for: Qualitative assessment of providers’ experiences with a segmentation counseling tool for family planning in Niger
Source: Reprod Health. 2023 May 10;20:71. doi: 10.1186/s12978-023-01617-9 (PMC10170744; doi:10.1186/s12978-023-01617-9)
Supplement: Supplementary file 5 — Additional file 5. Figure 3. Data collection plan for qualitative study in the intervention arms, in Dosso region. [file 12978_2023_1617_MOESM5_ESM.docx]

**Figure 3.** Data collection plan for qualitative study in the intervention arms, in Dosso region

**Conduct one IDI in 8 CSIs** in Doutchi, Dosso and Loga

**Intervention Arms**

Arm 1

(15 CSI)

16 IDIs total

Segmentation began in 2017 under IMPACT 1

**Conduct one IDI in 8** **CSIs** in Doutchi, Boboye and Tibiri

Arm 2

(15 CSI)

Segmentation began in late 2019

Abbreviations: IMPACT - Initiative de Mobilisation pour l'Accès à la Contraception pour Tous; CSI - Centres de Santé Integré; IDI - In-depth Interview
